# Supplementary material for: Resolving the full spectrum of human genome variation using Linked-Reads
Source: Genome Res. 2019 Apr;29(4):635–45. doi: 10.1101/gr.234443.118 (PMC6442396; doi:10.1101/gr.234443.118)
Supplement: Supplemental Material [file supp_gr.234443.118_Supplemental_longranger-2.2.zip › longranger-2.2.tar.bz2!/lr2.2-public/lib/stan/cmdstan/stan/doc/api/html/agrad_2fwd_2functions_2is__inf_8hpp_source.html]

Stan: src/stan/agrad/fwd/functions/is\_inf.hpp Source File


|  |  |
| --- | --- |
|  | Stan  2.5.0  probability, sampling & optimization |


- Main Page
- Related Pages
- Namespaces
- Classes
- Files

- File List
- File Members

All Classes Namespaces Files Functions Variables Typedefs Enumerations Enumerator Friends Macros Pages

- src
- stan
- agrad
- fwd
- functions

is\_inf.hpp

Go to the documentation of this file.

1 #ifndef STAN\_\_AGRAD\_\_FWD\_\_FUNCTIONS\_\_IS\_INF\_HPP

2 #define STAN\_\_AGRAD\_\_FWD\_\_FUNCTIONS\_\_IS\_INF\_HPP

3

4 #include <stan/agrad/fwd/fvar.hpp>

5 #include <stan/math/functions/is\_inf.hpp>

6

7 namespace stan {

8

9  namespace agrad {

10

19  template <typename T>

20  inline

21  int

22  is\_inf(const fvar<T>& x) {

23  using stan::math::is\_inf;

24  return is\_inf(x.val());

25  }

26

27  }

28 }

29

30 #endif

stan::agrad::fvar

**Definition:** fvar.hpp:13

stan::agrad::fvar::val

T val()

**Definition:** fvar.hpp:18

fvar.hpp

stan::agrad::is\_inf

int is\_inf(const fvar< T > &x)

Returns 1 if the input's value is infinite and 0 otherwise.

**Definition:** is\_inf.hpp:22

stan::math::is\_inf

int is\_inf(const double x)

Returns 1 if the input is infinite and 0 otherwise.

**Definition:** is\_inf.hpp:19

is\_inf.hpp


---

[ Stan Home Page ]

*© 2011–2014,
Stan Development Team.*
